# Gastroduodenal Bleeding and Perforation in Diabetic Patients with Metabolic Syndrome (The Results of a 15-Year Observation of City Residents with Intensive Urbanization)

**Published:** 2019-10

**Authors:** Marat IMASHEV, Alexandr FURSOV, Bagdat IMASHEVA, Roman FURSOV, Yerzhan KUSPAEV, Timofei KOVALENKO, Alishan ISMAILOV, Aigul ABDULDAYEVA, Irina VOLCHKOVA

**Affiliations:** JSC 'Astana Medical University', Astana, Kazakhstan

**Keywords:** Metabolic syndrome, Gastroduodenal bleeding, Diabetes mellitus

## Abstract

**Background:**

The prevalence of bleeding and perforation against the diabetes mellitus, obesity and metabolic syndrome (MetS) is studied not sufficiently.

**Methods:**

The period of collecting the material was 15 years (2003–2017). The observation analysis was conducted for the patients at polyclinics observations, by the doctors of first-aid, in the surgical departments of the Astana City, Kazakhstan. The number of first-aid visits to patients, the medical cards of the patients with gastroduodenal perforation (GDP) and gastroduodenal bleeding (GDB) were analyzed.

**Results:**

The rate of annual growth of indices of overall morbidity Rgm=1.0%, obesity in combination with diabetes was Ro=1.7%; and morbidity with metabolic syndrome was Rm=3.1%. The diagnosis of GDP was revealed in 0.63 men with MetS for every 100000 urban people. The diagnosis of GDB was registered in 2.12 men for 100000 urban people. The index of the annual growth in patients with MetS had the tendency to the growth (Rms=3.1%).

**Conclusion:**

The high medical and social significance of diseases of the digestive system among the adult population in Kazakhstan is determined by the annual increase in the incidence rate and a clear decrease in the quality of life of such patients, which necessitates the search for scientifically based ways to improve medical care for this population.

## Introduction

Among patients with ulcerative bleeding, the proportion of people suffering from diabetes mellitus (DM) and obesity is about 10%. At the same time, the amount of patients with diabetes in combination with acute gastroduodenal bleeding is actually more than it is commonly believed (1). Mortality of diabetic patients with bleeding is higher than in persons without diabetes for 1.6 times, and mortality with gastroduodenal perforation was for 1.7 times (2). This problem determines the importance and necessity to study the effect of diabetes on the organs of the gastroduodenal zone.

In the Republic of Kazakhstan (RK) the following are needed to investigate: a) the frequency of erosive and ulcerative lesions of the stomach and duodenum in diabetic patients in combination with obesity and metabolic syndrome; b) the prevalence of combination of diseases among different layers of the urban population; c) the statistics of asking for help of the ambulance service due to erosive and ulcerous perforation and bleeding in this category of patients.

Ten years' experience carried out by us shows this pathology is the most prevalent disease among the adult inhabitants in our region. Frequent complications, especially ulceral bleeding proves about the fact that the majority of the patients does not receive adequate medicamentose therapy which is the only method providing the unrecurrence course of the disease.

All these aspects served as the base for work on the organization of the surgical service in gastroduodenal bleeding and perforation in diabetic patients with metabolic syndrome.

We aimed to research the prevalence of surgical gastroduodenal complications (bleeding, perforation) against diabetes mellitus in people with obesity and metabolic syndrome.

## Methods

The study was conducted in the following directions: polyclinic observation of patients (at the level of primary outpatient care), analysis of statistical data of emergency medical care and hospital studies (in surgical departments). For obtaining the information about the incidence rate among the urban population targeted screening was conducted in various regions of Astana City, Kazakhstan.

The results were taken into account both in absolute figures and per 100000 people of the population. For the reliable demographic picture, the dynamics of demographic growth in Astana city and its relation to the health indicators of the population were studied. The statistics of calls of doctors of the ambulance station concerning gastroduodenal bleedings and ulcerative perforations was investigated. The frequency of hospitalizations of patients to surgical hospitals by ambulance brigades and statistics of visiting patients by polyclinic doctors was analyzed. The work of the outpatient gastroenterological center (as a pilot project) in the observation and treatment of these patients was studied.

The diagnosis of "obesity" was put after the measurement of BMI, if the body mass index (BMI) was≥30 (kg/m2) according to WHO recommendations. Metabolic syndrome (MetS) was diagnosed in accordance with international recommendations of the International Diabetes Federation (2006). For the assess of the concomitant pathology, the indices of comorbidity CI (Charlson Index) (3) and KF (Kaplan-Feinstein) were used (4).

The degree of hemorrhage was calculated by clinical and laboratory criteria. On the base of glycemia, glucosuria and ketonuria indices the level of compensation of carbohydrate metabolism in diabetes mellitus (compensation, subcompensation, and decompensation) was determined. To determine the activity of bleeding and evaluation of hemostasis, the endoscopic criteria on Forrest J.A. (5) was taken into account. The endoscopic picture of the mucous membrane of the body of the stomach was divided into 4 types (6). As a control group 150 people without diabetes, obesity or metabolic syndrome were selected (for comparison with a normal endoscopic picture and for instrument calibration). The age and sex composition of the patients in the control group were similar to the main one. The collection period is 15 years (2003–2017). The main volume of research and treatment (up to 70%) was conducted at the clinical bases of the Department of General Surgery. The rest (about 30%) of the examinations and hospital treatment was conducted with the participation of employees of other surgical hospitals of Astana City.

According of ethic characteristic of the research with international requirements was considered in the Committee of Ethics of Astana Medical University.

## Results

At first, urban urbanization indicators in the RK were analyzed. The city of Astana was chosen, which showed the most intensive population growth. It was the leader of growth, both in Kazakhstan and in Central Asia (7). The number of residents in Astana from Jan 2003 to Dec 2017 grew almost two times: from 501998 to 1,029,556 people. Our calculations confirm that the annual rate of population growth (R%) reaches the level of 7.01%. According to the results of the statistics of all appeals to the polyclinics of the city (all of them perform primary medical–sanitary aid), the prevalence of peptic ulcer among the population (10.98%) was determined. The growth of the average incidence rate of the urban population of the RK with gastroduodenal ulcers and erosions has been revealed - from 80.3 to 106.2 (the number of newly diagnosed cases, per 100000 people). In Astana, this indicator increased from 91.2 to 108.7, with an annual growth rate (Ru%) of 1.3% (Table 1).

**Table 1:** Morbidity of Peptic Ulcer Disease, Diabetes, Obesity and Metabolic Syndrome in Kazakhstan

***Category of population***	Stomach ulcer and duodenum*			Diabetes*			Obesity/MetS*		
	***2003***	***2017***	***Ru***	***2003***	***2017***	***Rd***	***2003***	***2017***	***Ro/Rms***
Astana	91.2 (2.0)	108.7 (2.1)	1.3	150.3 (2.2)	178.8 (2.1)	1.3	87.3/23.9 (1.1)	109.1/37.5 (1.0)	1.7/3.8
Urban population of Kazakhstan	80,3 (2.1)	106,2 (2.0)	2.2	188.5 (2.1)	236.2 (2.2)	1.7	78.4/ 22.1 (1.1)	96.9/ 36.7 (1.0)	1.6/ 4.4
Women	35.2 (1.1)	48.0 (1.1)	2.4	185.5 (2.1)	273.1 (2.2)	3.1	112.3/ 35.5 (1.1)	138.5/ 63.2 (1.1)	1.6/ 5.9
Men	147.2 (2.1)	169.4 (2.0)	1.0	115.1 (2.2)	84.5 (2.2)	−1.8	89.5/ 23.0 (2.2)	95.3/ 30.3 (2.2)	0.4/ 2.1
Patients 18 yr and older	81.1 (2.2)	112.2 (2.2)	2.6	200.5 (2.2)	327.3 (2.2)	4.2	225.9/ 56.0 (2.2)	253.1/ 78.2 (2.2)	0.8/ 2.6
Patients 15–17 yrold	8.3 (0.01)	10.3 (0.01)	1.6	11.5 (0.01)	16.8 (0.01)	3.1	12.9/ 3.0 (0.01)	15.8/ 3.1 (0.01)	1.5/ 0.2
M	99.0 (2.2)	101.0 (2.2)	0.1	126.1 (2.2)	201.4 (2.2)	4.0	73.8/ 23.1 (2.2)	92.2/ 33.8 (2.2)	1.7/ 3.1

* - number of diseases SD, registered for the first time, per 100 000 people of the population with a confidential interval of 95% and a statistical significance level, *P*≤0.05;

**(…)** – SD; MetS - Metabolic Syndrome; *R*…% - the annual increase: Ru – ulcer;

Rd - diabetes; Ro – obesity; Rms - metabolic syndrome; *M* - the average for country

The incidence of diabetes in the RK in 2017 was 178.8 (the number of newly diagnosed, per 100000 people, SD 2.1; *P*≤0.05). Out of this number of patients with diabetes, the average incidence of obesity in the RK was identified, which is 92.2 (SD 2.2; *P*≤0.05). In the city of Astana this indicator was higher and reached the level of 109.1 (the number of newly identified, per 100000 people), with the annual growth rate (Ro%) for the last 15 years at 1.7% (Table 1). The reported rates of obesity (Ro%) significantly outperform the overall morbidity (R general morbidity%) for Kazakhstan (Ro=1.7 vs. Rgm=1.0). The overall incidence was calculated as an integrated indicator for all disease classes listed in ICD-10.

The highest rates of annual growth in the RK were detected in the metabolic syndrome, much less the rate of increase in obesity and the overall morbidity (Rms=3.1 vs. Ro=1.7 vs. Rgm=1.0). Especially high rates were in women (Rms=5.9).

The study of medical records of 13756 patients showed that they were all delivered in urgent order to three multi-profile city hospitals. Out of them with perforations – 9366, with gastroduodenal hemorrhage – 4390. Their age varied from 15 to 90 years. The prevalence is in elderly patients from 60 to 74 yr – 5732 people (41.67%). People of middle age from 45 to 59 yr – 3457 people (25.13%). The age group from 75 to 90 yr was 3212 people (23.35%). Patients of young age from 15 to 44 yr were 1355 people (9.85%).

The main condition for sending patients to the hospital was standard. If the medical station staff after the examination of the patient ascertained a preliminary diagnosis or "gastroduodenal perforation - GDP" or "Gastroduodenal bleeding - GDB", then such a patient was immediately transported to a surgical hospital for excluding or confirming the diagnosis. In total, from 2003 to 2017 in Astana, there were registered 13756 such cases. The average per year is 917 cases. Out of them, the average annual number of calls for NSRs diagnosed with GDP was 624.4 (MD 596.0, SD 163.7, Confidence interval Student – 0.205 at α=0.9), and the average annual number of calls with GDB diagnosis was 292.7 (MD 269.0, SD 89.9, Confidence interval Student – 0.164 at α=0.9).

The average annual rate of hospitalization of patients in surgical clinics with the diagnosis of GDP was 36.81 per 100000 people. (MD 37.74, SD 7.26, Confidence interval Student – 0.10 at α=0.9). With obesity and diabetes – 2.48 per 100000 (MD 2.48, SD 0.41, Confidence interval Student – 0.05 at α=0.9). Out of them, with the metabolic syndrome – 0.63 (MD 0.63, SD 0.1, Confid. Interval Student – 0.05 at α=0.9).

Correlation (r) between indicators of patients delivered to surgery with suspicion of GDP and GDB with diabetes and obesity is 0.99 (Pearson). The correlation (r) between the indices of hospitalized patients in surgery with diabetes and obesity on the sing of perforation and bleeding was slightly lower – 0.92 (Pearson). The received results on hospitalization of patients with obesity and diabetes mellitus in surgical hospitals are presented in Table 2.

**Table 2:** The number of admitted and hospitalized patients with surgical gastroduodenal complications with obesity and diabetes*

***Year***	***Population number***	Admitted by emergency to the hospital*		Patients hospitalized into surgical department*			
		***GDP***	***GDB***	***GDP***		***GDB***	
		***Average***	***Average***	***Average***	***With D-O/MetS***	***Average***	***With D-O/MetS***
2003	501998	81.275	39.841	40.040	3.22/0.81	43.426	6.51/1.76
2004	510533	82.659	41.329	42.700	3.01/0.76	42.7	6.6/1.72
2005	529335	86.901	36.461	45.718	3.01/0.76	45.718	6.69/1.74
2006	550438	87.203	36.335	44.510	2.88/0.73	43.602	6.59/1.73
2007	574448	89.651	38.820	43.868	2.76/0.70	41.779	6.63/1.72
2008	602684	90.926	38.494	42.477	2.63/0.67	39.822	6.69/1.87
2009	605254	91.201	39.653	43.122	2.52/0.64	39.653	7.41/2.06
2010	649146	91.813	41.439	37.742	2.49/0.63	36.972	7.63/2.04
2011	697156	92.662	43.893	35.573	2.31/0.59	34.426	7.81/2.33
2012	742918	92.742	43.881	35.132	2.22/0.56	30.017	8.02/2.24
2013	778083	89.836	44.982	33.030	2.09/0.54	30.845	8.41/2.46
2014	811529	92.788	43.252	30.190	2.03/0.52	54.835	8.58/2.48
2015	861968	92.579	45.825	28.423	2.04/0.53	41.301	8.59/2.58
2016	905368	93.001	46.390	27.061	2.02/0.52	45.506	8.59/2.57
2017	1029556	93.049	46.039	22.534	2.01/0.51	44.679	8.62/2.58
Mean		89.88	41.77	36.81	2.48/0.63	41.01	7.56/2.12
R (%)	7.01	0.97	1.04	−2.91	−2.51/−2.47	0.19	2.16/3.1

* Values were calculated on 100000 people, urban;

O– Obesity; D – Diabetes; MetS - Metabolic Syndrome; GDP – gastroduodenal perforation; GDB – gastroduodenal bleedings; R (%) **–** Annual increase in %

Thus, the number of SMP calls in connection with the growth of the population of Astana is constantly increasing. If in 2003 there were 152018 of them, in 2009 – 223336, then in 2017 the number of calls to the NSR and home visits (respectively, and the number of recorded diagnoses) already exceeded 270000. Based on this data, the annual rate indicator was calculated the increase in the number of emergency examinations by the doctors of the SMP - Emergency Medical Examination (Re%). During the 15-year period, Re was 5.17%. Comparative analysis revealed that in Astana the index of SME calls per capita is higher than the average annual rate for Kazakhstan (0.45 vs. 0.4). Hospitalization of patients was carried out mainly by paramedic brigades (66%). The proportion of doctors accounted for 21%. In especially severe cases, hospitalization was performed by specialized medical or anti-shock teams (13%). The percentage of coincidence of diagnoses after the surgeon's examination in the hospital was higher in the medical and specialized teams of the medical help station. Significantly lower is in the feldsher brigades, because of hyper diagnostics.

All patients taken to the hospital's casualty ward had severe pain symptoms, simulating acute surgical complication of peptic ulcer. As the analysis showed, in 75% of cases prompted medical personnel of the NSR to make a decision to transport these persons to the surgical department. In the remaining 25% of cases, the decision was made because of doubts about the diagnosis. Out of the total number of patients delivered to the admission ward of the hospital with suspicion of GDP, only 3831 (40.9%) were hospitalized for surgery. Out of them, with obesity and diabetes were only 257 (6.7%), and with the metabolic syndrome were even less – 65 (1.7%). With suspicion of GDB 4311 (98.2%) were hospitalized. Out of them with obesity and diabetes were 794 (18.4%), with the metabolic syndrome there were 224 (5.2%). In other cases, the diagnosis of GDP and GDB has not been confirmed. If the endoscopic picture confirmed an erosive or ulcerative lesion of the mucosa, but the active bleeding stopped, and the patient did not need surgical help, he was hospitalized into the gastroenterological department.

Our statistical calculations determined:

- With a diagnosis of GDP combined with diabetes and obesity, 248 people were hospitalized into a surgical hospital for every 100000 inhabitants of the city. Out of that 25.4% had clinical signs of MS. Only 0.63 people per 100000 inhabitants of the city after the examination in the surgical department confirmed the diagnosis of GDP;
- With the diagnosis of GDB in combination with diabetes and obesity, every 7.56 people per 100000 urban population were hospitalized. Clinical signs of MS were revealed in 28.0%. We revealed that in the recalculation for 100000 residents of the city of Astana, only 2.12 people were diagnosed with GDB.
- Moreover, the annual increase in individuals with MS and bleeding is very high, (Rms%=3.1). At the same time, the growth rate of gastroduodenal perforations in individuals with MS had negative values (Rms%=−2.47). This shows only one thing, the number of these complications decreases from year to year. Further, 70% of patients delivered to the hospital (3–6 h after the onset of the pain syndrome) with GDP had purulent peritonitis. The operation of suturing of perforation was performed in 75% of cases, and suturing with vagotomy and pyloroplasty - in 25%. Up to 65% of all operations were performed using median laparotomy, 35% were performed laparoscopically.

With gastrointestinal hemorrhage, only 8% persons were operated on. Sixteen operations were performed: 2 gastrectomies (later gastric cancer was detected histologically); 2 suturing of bleeding ulcer, 12 suturing of varicose veins of the esophagus after multiple endoscopic hemostasis (chipping, plasma and electrothermocoagulation, clipping). Among all persons taken to the GDB hospital, 67.9% had ulcers and erosions. In the remaining cases, there was a combination of ulcerative mucosal damage with Mallory-Weiss syndrome, Dyulafoy's defeat was in 13.0%, bleeding polyps in 4.1%, stomach cancer in 2.2%, bleeding from varicose-extended Esophageal veins – 12.8%. The distribution of patients by the nature of the source of bleeding shows that in diabetes mellitus and the metabolic syndrome the structure of the causes of gastroduodenal bleeding changes, the severity and proportion of acute erosive-ulcerative lesions increase (Table 3).

**Table 3:** Characteristics of GDB hospitalized patients with value of comorbidity and risk of recurrence from ulcer bleedings

***Variable***	***Patients D and O n=794***	***Risk of bleeding***		***Patients* MetS n=224**	***Risk of bleeding***	
		***low (IIb-III)***	***high (Ia-IIa)***		***low (IIb-III)***	***high (Ia-IIa)***
Comorbidity Charlson						
Score low (0)	402 (50.6)	354(88.1)	48(11.9)	64(28.6)	31(48.4)	33(51.6)
Score medium (1–2)	239 (30.1)	165(69.0)	74(31.0)	95(42.4)	40(42.1)	55(57.9)
Score high (>2)	153 (19.3)	104(68.0)	49(32.0)	65(29.0)	22(33.8)	43(66.2)
Kaplan -Feinstein						
Score (0)	127(16.0)	87(68.5)	40(31.5)	14(6.3)	7(50.0)	7(50.0)
Score (1)	250(31.5)	132(52.8)	118(47.2)	36(16.1)	19(52.8)	17(47.2)
Score (2)	236(29.7)	105(44.5)	131(55.5)	62(27.7)	25(40.3)	37(59.7)
Score (3)	181(22.8)	80(44.2)	101(55.8)	112(50.0)	37(33.0)	75(67.0)
Anamnesis and treatment						
Previous uncomplicated peptic ulcer disease	127(16.0)	52(40.9)	75(59.1)	59(26.3)	26(44.1)	33(55.9)
Use of drugs associated with peptic ulcer bleeding	651(82.0)	301(46.2)	350(53.8)	168(75.0)	81(18.2)	87(51.8)
Use of antiulcer drugs	201(25.3)	102(5.7)	99(49.3)	58(25.9)	32(55.2)	26(44.8)

* n - number; (%); O – Obesity; D – Diabetes; MetS - Metabolic Syndrome; GDB – gastroduodenal bleedings;

Among all patients with diabetes mellitus with acute gastroduodenal bleeding, a light degree of severity of diabetes was stated only in 19.0% of patients, average in 43.2%, severe in 37.8% of cases. Out of all patients, patients with decompensated diabetes mellitus and metabolic syndrome were predominated. The condition of compensation was diagnosed in 21.2% of patients, subcompensation in 14.8%, in decompen-sated state there were 64.0% of patients.

## Discussion

Data on the prevalence of diabetes, obesity and metabolic syndrome among the population of Kazakhstan are quite contradictory. This is due to the lack of large-scale studies in remote regions of the country. Single publications on the study of the prevalence of patients with obesity give statistics of past ten year'speriod (8). For example, spatial studies of the incidence of obesity were conducted for the first time (9). In 2008 it was Pavlodar region (143.0 per 100000 population) and Mangystau region (139.7), in the third-place Astana (135.5). However, now the process of spreading the diseases occurs differently, against the backdrop of intensive migration of the population from the periphery to big cities. Significantly increased the number of urban residents (both healthy and sick), especially over the past 10 years (7). There is convincing evidence that among the obese people the greatest number of requests for medical care is registered (10). According to the result of our work, the cities of Almaty, Pavlodar, and Astana are classified as cities with a high extensive obesity index of 13.7%. The lowest extensive obesity indices are in Atyrau Region (2.2%). Moreover, we found out that the majority of examined with obesity does not know his or her exact weight and height. An attempt was made to systematize and correct statistical data in the RK on diabetes and obesity (11). However, questions related to ulcerative gastroduodenal complications in this group of patients have not been studied. Having studied the world literature, scientific electronic databases available on the Internet in turned out that this problem received insufficient attention, especially in the comparative context of "diabetes-obesity-MetS". Similar studies as well as in our case indicated a rapid spread of obesity and MetS among the urban population especially among women (12). The risk of metabolic disorders in some African men is associated with their high socioeconomic status. And fatness and obesity in women is a consequence of cultural heritage, which increases the risk of MetS. In Israel, on the contrary the prevalence of metabolic syndrome was higher in men than in women (13). The classical triad of perforation is so typical that it is impossible to make a mistake (14). Individuals born in Israel had lower prevalence of metabolic syndrome (9.7%) than those born in other countries (13.3%–15.0%). In the United States, one in ten teenagers with metabolic syndrome is identified (15). Prevalence was higher in males than females (13.0% vs. 6.4%, *P*<0.05).

For example, the rural population of China recorded a range from 11.5% to 54.2%. In some countries, obesity and MetS are relatively low (2.3%), while the prevalence of diabetes is 39.1% in men (16). As for the development of gastroduodenal pathology in this category of people, Helicobacter pylori infection increases the risk of MetS, diabetes and complications (17). There are studies in which atypism of clinical symptoms is indicated in patients with diabetes, obesity and MetS (18).

As a result, the prevalence of these surgical complications among the urban population with diabetes, obesity and MetS, the frequency of hospitalization of patients with bleeding and perforation, the comparison of comorbid disorders with the endoscopic picture, etc. are still not sufficiently studied.

## Conclusion

The studied indicators could be considered as one of the criteria for predicting the pathological process, as well as the effectiveness of methods of prevention (or treatment), since the above showed high prognostic significance and objectivity when applying the developed methodological approach to the study of indicators quality of life of surgical patients in the pre- and postoperative periods.

## Ethical considerations

Ethical issues (Including plagiarism, informed consent, misconduct, double publication and/or submission, redundancy, etc.) have been completely observed by the authors.
